# Impact of EVOH, Ormocer^®^ Coating, and Printed Labels on the Recyclability of Polypropylene for Packaging Applications

**DOI:** 10.3390/polym17243332

**Published:** 2025-12-17

**Authors:** Romana Schmiedt, Michael Krainz, Katharina Tosic, Farshad Sharbafian, Simon Krauter, Victoria Krauter, Martin Novak, Bernhard Rainer, Michael Washüttl, Silvia Apprich

**Affiliations:** 1 Institute of Packaging and Resource Management, Department Applied Life Sciences, University of Applied Sciences, Favoritenstraße 222, 1100 Vienna, Austria; romana.schmiedt@hcw.ac.at (R.S.); katharina.tosic@hcw.ac.at (K.T.); farshad.sharbafian@hcw.ac.at (F.S.); victoria.krauter@hcw.ac.at (V.K.); martin.novak@hcw.ac.at (M.N.); silvia.apprich@hcw.ac.at (S.A.); 2 OFI-Österreichisches Forschungsinstitut für Chemie und Technik, Franz-Grill-Straße 5, Obj. 213, 1030 Vienna, Austria; michael.krainz@ofi.at (M.K.); michael.washuettl@ofi.at (M.W.)

**Keywords:** plastic recycling, plastic packaging, polypropylene, ethylene-vinyl alcohol, labels, recyclability, Ormocer^®^

## Abstract

Flexible packaging often consists of multilayer films that combine different materials to achieve high barrier performance, but these structures are incompatible with current recycling technologies. Polyolefins such as polypropylene (PP) offer more recyclable alternatives but require additional oxygen-barrier materials that do not compromise recyclability. This study investigates the influence of ethylene vinyl alcohol (EVOH), Ormocer^®^ barrier coating, and PP labels with different adhesives on PP recyclability. Recyclates were produced using twin-screw extruder to simulate the recycling process and then injection-molding to make tensile test specimens. Mechanical properties, melt flow rate (MFR), oxygen induction time (OIT), and odor were evaluated. Findings showed that low label content (5–12.5%) has minimal impact on recyclate quality. The addition of 10% EVOH increased the elastic modulus of PP granulate and cast-PP (cPP) film by 26% and 14%, respectively, and improved oxidation stability by 9%, while reducing cPP film impact strength by 77%. Ormocer^®^ decreased mechanical performance, particularly elongation at break (−18%), likely due to defect-inducing particles, but had limited influence on MFR. Labels and Ormocer^®^ also introduced odor variations. Overall, the findings indicate that EVOH up to 10% and labels up to 12.5% yield promising results, providing guidance for designing recyclable, monomaterial packaging.

## 1. Introduction

Known for its lightweight, material efficient design, flexible film packaging protects a wide range of products and is particularly valued in the food industry for its ability to adapt to diverse shapes and sizes. In 2018, the annual demand for flexible films in Europe was estimated to be between 13 and 15 million tons (MT) [1]. Of this total, polyethylene (PE) was the most widely used material, accounting for approximately 8.5 to 9 MT. Meanwhile, polypropylene (PP) and multilayer films each comprised between 2 and 2.5 MT [1].

Multilayer packaging films are a critical component of the flexible packaging industry due to their ability to combine the functionalities of multiple materials into a single structure. Unlike mono-materials, which often fall short in simultaneously meeting all functional requirements, multilayer films offer effective barriers against water vapor and oxygen, protection from foreign flavors, and a combination of flexibility and strength, while maintaining a low packaging weight [2,3]. This makes them advantageous for sensitive or premium foods such as convenience meals, meat, dairy products, and high-quality snacks [4].

Despite advantages in product containment and protection, the use of different materials in multilayer structures complicates the recycling process. Currently, it is not possible to identify and segregate the layers using standard plastic recycling technologies [5,6]. Consequently, in European countries, multilayer packaging is commonly incinerated with energy recovery [7]. The EU’s new Packaging and Packaging Waste Regulation (PPWR) mandates that by 2030, all plastic packaging introduced to the EU market must be either reusable or recyclable in an economically viable manner [8]. This regulatory focus puts additional pressure on industry to find recyclable solutions that do not compromise packaging performance.

Polyolefins such as PP and PE, which, as previously mentioned, already constitute a substantial portion of the flexible film market, are strong candidates for replacing multilayer flexible food packaging. These mono-materials are better recyclable, and their widespread use would help reduce overall material variability while leveraging well-established manufacturing processes. However, additional barrier properties must be incorporated, which could be achieved through methods such as applying coatings, metallization, or the use of AlOx/SiOx layers or EVOH, provided these materials are used within acceptable limits [9]. In particular, the oxygen-barrier properties require considerable improvement, while the moisture barrier properties of PP and PE are already sufficient [10]. In our study, we focus on two of the strategies to improve the barrier of polypropylene, namely OROMOCER^®^ as an example of a coating as well as EVOH.

Ethylene vinyl alcohol (EVOH), is a flexible, clear, thermoplastic material with excellent oxygen-barrier properties [11,12]. These characteristics make EVOH an attractive option for enhancing the barrier performance of polyolefin-based packaging while using the smallest possible amount of EVOH to balance barrier effectiveness and recyclability. However, current guidelines for assessing the recyclability of plastic packaging vary regarding the permissible amount of EVOH in flexible packaging. For instance, RecyClass gives flexible PE or PP films containing less than 5% EVOH content a “conditional–limited compatibility” rating, meaning it slightly impacts the recycling process and/or the quality of the recyclate [13]. Cyclos-HTP, however, considers EVOH as a category 2 impurity that is “conditionally compatible” in terms of recyclate application) but does not specify any permitted amount [14]. Meanwhile, CEFLEX states that a maximum of 5% EVOH, relative to the total packaging structure weight, is compatible with PE or PP mechanical recycling [15]. These differences highlight the need to clarify how varying EVOH contents affect PP recyclate quality and how these findings align with current recycling guidelines.

In exploring coatings to enhance the oxygen-barrier properties of polyolefin monomaterials, Ormocer^®^ materials have emerged as promising candidates. These coatings provide excellent oxygen and moisture barrier performance, along with durability, flexibility, and strong adhesion to polymeric substrates [16,17,18,19]. Ormocer^®^s, or organically modified ceramics, are a trademark of the Fraunhofer-Gesellschaft ISC and are characterized by a hybrid composition that combines the properties of inorganic ceramics and organic polymers [20]. Typically applied in liquid form, Ormocer^®^ coatings are transparent and only a few micrometers thick [21]. More recently, their potential use in food packaging has been investigated [22]. The adoption of Ormocer^®^ in food packaging applications is influenced not only by their performance but also by their recyclability, as Ormocer^®^ coatings are relatively expensive. However, if Ormocer^®^ coatings enable effective recycling, their environmental advantages could outweigh the higher initial costs. According to Emmert et al., a thin Ormocer^®^ coating is assumed to be compatible with mechanical recycling [22]. Yet, no particular studies have been found that investigate the recyclability of Ormocer^®^ and its influence on recycling performance.

Labels play a crucial role in providing essential product information to consumers, including product identification, nutritional content, ingredient listings, net weight, and manufacturer details [23]. Additionally, packaging labels can support recycling efforts by providing clear disposal instructions, incorporating digital watermarks, or utilizing chemical tracers [24]. For example, the Holy Grail 2.0 project has explored digital watermarks—imperceptible codes on packaging—detectable by a high-resolution camera to improve the precision and efficiency of sorting processes [25]. A wide range of labeling technologies and methods are currently available on the market, with pressure-sensitive labels being a cost-effective and widely used solution.

The removal of labels presents advantages for recycling but may be detrimental if labels are used for sorting, e.g., if they contain tracers [26]. An alternative approach is to manufacture labels from the same material as the packaging, such as PP labels for PP packaging or PE labels for PE packaging. In this case, the labels may remain attached to the base material during recycling, thus simplifying the process. Nevertheless, adhesives used in such labels must have minimal impact on the recycling process or the quality of the recyclate. Since labels are typically printed, they are generally only recyclable within the colored stream, and the amount of color used in labels should be kept to a minimum.

RecyClass guidelines generally consider adhesives compatible only when they are removable by washing, making water-soluble or water-releasable systems preferable [27,28]. While some commonly used pressure-sensitive and hot-melt adhesives show limited impact on recycling, other adhesive types require further evaluation. Importantly, the RecyClass guideline focuses on rigid HDPE packaging, and the influence of permanent label adhesives on PP flexible packaging remains largely unknown, highlighting the need for additional research.

**Figure 1:**
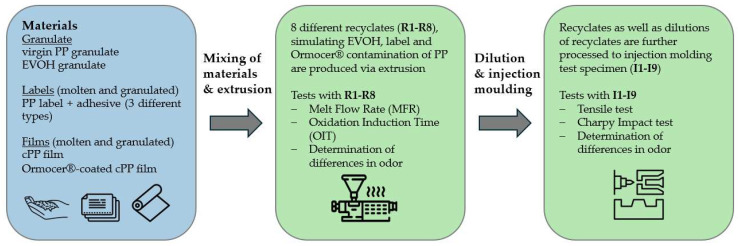
Schematic overview of the processing of the materials and the tests performed after each step indicated by arrows. (Icons: Flaticon.com).

There are only few experimental studies available on the recyclability of label and EVOH concentrations along with key recycling parameters. Similarly, the recyclability of Ormocer^®^ has mostly been assumed theoretically and rarely tested in actual mechanical recycling processes. Therefore, this study investigates the influence of labels and adhesives, along with barrier materials such as EVOH and Ormocer^®^ coating, on the mechanical properties of recycled PP. Additionally, processing parameters such as melt flow rate (MFR) and oxygen induction time (OIT) are examined, and differences in odor were analyzed in order to assess the feasibility and potential consequences of using high proportions of recycled material. A schematic overview of the workflow is presented in Figure 1. The influence of EVOH was examined on both cast polypropylene (cPP) films and virgin PP granulates, as films and granulates as a source material can exhibit different behaviors during recycling processes. Labels and adhesives were studied exclusively with granulates, as this approach is more general and accounts for both rigid and flexible packaging recycling. Ormocer^®^ coating, on the other hand, was evaluated only on pre-coated films. Since Ormocer^®^ is applied directly as a coating on films and is not present in the granulate form, its impact is best studied at the film level, aligning with its intended use in coated flexible packaging. To analyze the mechanical properties, tensile test and Charpy Impact test specimens were produced via injection molding. To study the effects of varying concentrations of EVOH and labels, dilution was carried out during this stage.

## 2. Materials and Methods

### 2.1. Barrier Materials

In this study, the ORMOCER^®^ material CBS004, a three-component inorganic–organic hybrid polymer with a solids content of 30%, was obtained from Fraunhofer ISC, Würzburg, Germany. EVOH granulate was provided by one of the project partners; however, due to confidentiality agreements within the project partners, no public datasheet was available, and disclosure of the exact grade or ethylene content is not permitted.

### 2.2. Polymer Granulates, Films and Label Materials

To assess the influence of EVOH, Ormocer^®^ as well as printed labels with various adhesives on the recyclate quality, the following materials were used:

- Polypropylene copolymer granulate virgin material (virgin PP granulate);
- EVOH granulate;
- Oriented polypropylene-label material, red/white colored, 52 µm thickness with solvent acrylate-based adhesive type 1 (PP label + adhesive type 1);
- Oriented polypropylene-label material, yellow/black colored, 52 µm thickness with rubber-based adhesive type 2 (PP label + adhesive type 2);
- Oriented polypropylene-label material, violet/black colored, 52 µm thickness with ultraviolet acrylate-based adhesive type 3 (PP label + adhesive type 3);
- Cast polypropylene film with 70 µm thickness (cPP film);
- Cast polypropylene film with a thickness of 70 µm, coated on a semi-industrial scale by Fraunhofer ISC with Ormocer^®^ CBS004. The coating was applied at a line speed of 5 m/min and subsequently cured at 100 °C for 20 s. (Ormocer^®^-coated cPP film).

**Figure 2:**
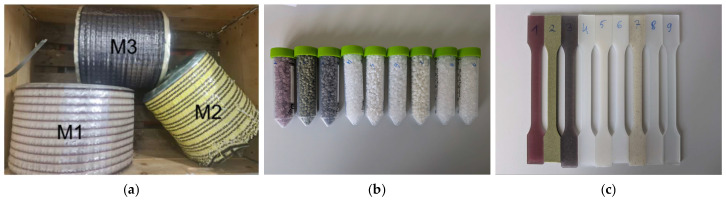
Label materials (**a**), recyclates as granulates as well as virgin PP granulate (**b**) and standardized injection-molding tension rods (**c**).

To simplify handling, the tests were not conducted on the labels themselves but rather on the leftover material remaining after the labels were punched out using punching grids (Figure 2a). EVOH granulate and virgin PP granulate were used as provided. The other materials were melted and granulated using a continuous agglomerator to render them processable for the following extrusion step. As mentioned earlier, according to the RecyClass design-for-recycling guideline, EVOH contents up to 5 wt% is considered as conditionally compatible with PP recycling. Therefore, EVOH content levels of 1%, 5% and 10% were selected to represent ideal, acceptable and challenging scenarios, respectively. Accordingly, label concentrations of 5, 12.5, 25, and 50 wt% were chosen to simulate increasing levels of label contamination during PP recycling. In this study, the dilution was carried out based on a mass percentage and 50 wt% label with 50 wt% PP granulate was assumed to represent an extreme worst-case scenario as a heavily decorated packaging material. In contrast, 5–25 wt% label content reflects more realistic levels of partial decoration commonly found in commercial packaging.

### 2.3. Production of Recyclates via Twin-Screw Extrusion

Twin-screw extruder Prism TSE 24 MC, with L/D ration of 40, maximum output 17 kg, was used to simulate the recycling process at a maximum temperature of 230 °C and a screw speed of 400 rpm. The detailed information and complete temperature profile applied during compounding are illustrated in Table S1.

Recyclates (R1–R8) were produced from materials listed in Section 2.1 according to the following mixing ratio:

- R1 (PP label 1): 50% PP label + adhesive type 1, 50% virgin PP granulate;
- R2 (PP label 2): 50% PP label + adhesive type 2, 50% virgin PP granulate;
- R3 (PP label 3): 50% PP label + adhesive type 3, 50% virgin PP granulate;
- R4 (PP granulate with EVOH): 90% virgin PP granulate, 10% EVOH granulate;
- R5 (cPP film with EVOH): 90% cPP film, 10% EVOH granulate;
- R6 (cPP film): 100% cPP film;
- R7 (cPP film coated with Ormocer^®^): 100% Ormocer^®^-coated cPP film;
- R8 (PP granulate): 100% virgin PP granulate.

In total, 8 different recyclates were obtained from the extrusion process in the form of pellets (Figure 2b). R9 corresponds to virgin PP granulate and is not an actual recyclate.

### 2.4. Production of Tensile and Charpy Impact Test Specimen via Injection Molding

Recyclates were further processed into test specimens for tensile test and Charpy Impact test via injection molding. Some recyclates were diluted with recyclate R8 or R6, respectively, before the injection-molding process. This was carried out in order to investigate the influence of the components at lower concentrations on the mechanical properties.

Recyclates R1–R4 were diluted with R8 (PP granulate) and recyclate R5 (cPP film with EVOH) was diluted with R6 (cPP film). Furthermore, virgin PP granulate as received was processed to test specimens via injection molding. Tension rods (ISO 3167, type A [29]) and bars (ISO 178 [30]) were produced using a Battenfeld Smart Power 25 injection-molding machine at a maximum temperature of 230 °C and injection pressure of 400 bar. The detailed information and complete temperature profile applied during injection-molding process is illustrated in Table S2. Examples of tension rods can be seen in Figure 2c. Table 1 summarizes the composition of the tensile test and Charpy Impact test specimen as well as denoting which recyclates were used for their production.

After the injection-molding process, the samples were stored for 1 week at 23 °C/50% relative humidity (RH) before further testing.

### 2.5. Method Description

#### 2.5.1. Tensile Test According to ÖN EN ISO 527-2 [31]

The elastic modulus, tensile strength and elongation at break were tested on 5 injection-molded tension rods (ISO 3167, type A) per test specimen on a Zwick tensile testing machine, type Z100 equipped with a 5 kN load cell, at a test speed of 50 mm/min.

#### 2.5.2. Impact Properties (Charpy) According to ISO 179-1 [32]

The test was carried out on 10 injection-molded bar (ISO 178) per test specimen on a Müller-Scherr Ges.m.b.H. & Co.KG, Linz, Austria, pendulum impact tester, type Frank 53302, with pendulum energies between 1 and 7.5 J depending on the sample type: 1 J for R1–R4; 2 J for R5; 4 J for 5a, 5b, R7; 7.5 J for R6.

#### 2.5.3. MFR According to ÖN EN ISO 1133-1/A [33]

The MFR tests were carried out on 10 granulate samples R1–R8 as well as virgin PP granulate at 230 °C and a weight of 2.16 kg using a MFR tester CEAST Sp:A, Italy, type 7026 measuring device.

#### 2.5.4. Determination of OIT, Dynamic by Means of Differential Scanning Calorimetry (DSC) According to EN ISO 11357-6 [34]

The OIT was determined on one granulate sample of R1–R8 as well as virgin PP granulate using a Mettler Toledo, USA DSC1/700 measuring device and a Mettler Toledo, USA XP 6 microbalance in a temperature range of 20 °C to 270 °C at a heating rate of 10 K/min with synthetic air purge gas (50 mL/min). The sample weight was approximately 10 mg in an aluminum test crucible, standard 40 µL with perforated lid. The evaluation was carried out at a 50 mW/g threshold for the extrapolated onset temperature.

#### 2.5.5. Determination of Differences in Odor According to DIN 10955 [35]

The granulates R1–R8 as well as virgin PP granulate were filled in closed glass containers, whereby a weight/volume ratio of 5 g to 100 mL was used and stored at room temperature in a dark environment for a period of 24 h. The injection-molding rods/bars were filled in closed glass containers, whereby a surface volume ratio of 6 dm^2^ to 600 mL was used and stored at room temperature in a dark environment for a period of 24 h.

The samples were then analyzed for differences in odor compared to room air, in accordance with DIN EN ISO 5495 [36], by asking 10 test persons to test sample pairs. Then they assessed the intensity of the difference in odor noticed between the sample and the reference (room air), on the basis of the categories given in Table 2. The minimum number of answers required for confirming significant differences was analyzed according to the relevant standard. After the assessment, the median of the differences in odor was calculated.

## 3. Results

### 3.1. Determination of Tensile Properties

#### 3.1.1. Impact of Labels on Tensile Properties

**Figure 3:**
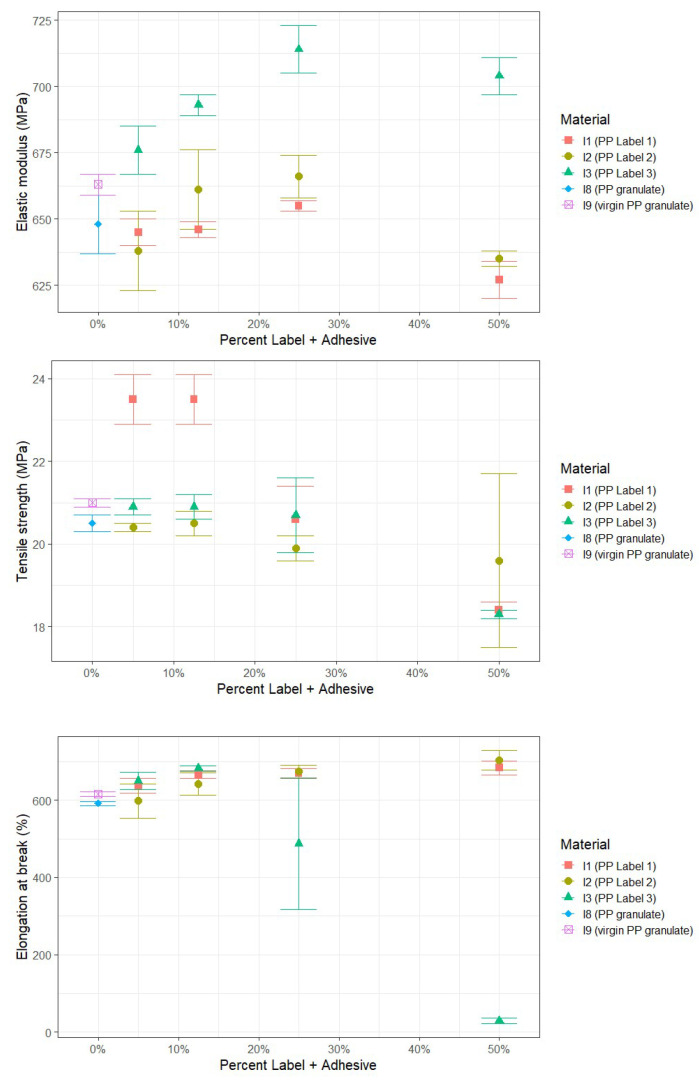
Results of tensile tests of PP containing varying content of labels after extrusion and injection molding.

The influence of labels on the mechanical properties of recycled PP was investigated by comparing samples with and without labels. Figure 3 presents the tensile test results for the samples with labels. Two materials without labels serve as references: I9 (virgin PP granulate) and I8 (PP granulate). I9 represents the virgin PP granulate material, which was directly processed into test specimens via injection molding. I8, on the other hand, refers to the virgin PP granulate material that was first extruded to form recyclate and then processed into test specimens through injection molding. The samples I1–I3, containing varying content of labels, underwent the same process as I8: they were extruded to form recyclates and then processed into test specimens via injection molding. Therefore, I8 (PP granulate) is a more relevant reference for comparison with I1–I3, as it shares the same processing history. However, I9 (virgin PP granulate) provides valuable insight into the impact of the extrusion step, which simulates the recycling process, on the mechanical properties of the material.

I8 (PP granulate) shows only slight reductions in mechanical properties compared to the virgin material I9, averaging a decrease of 2–4%. The influence of PP labels with varying color systems and adhesive types (acrylate, rubber, or UV acrylate based) on elastic modulus, tensile strength, and elongation at break is not discernible in samples with 5% and 12.5% PP label content compared to PP without labels. Notably, in the case of I1 (PP label 1), tensile strength is even higher than the reference. Labels with adhesive types 1 (acrylate-based) and 2 (rubber-based) do not exhibit any notable influence on the analyzed mechanical properties, even with a label content of 25%. However, lower average values for elastic modulus and tensile strength are observed for adhesive type 1 and 2 with a label content of 50% compared to I8 (PP granulate). The PP label with adhesive type 3 (UV acrylate-based) shows a reduction in elongation at break with 25% and 50% label content.

#### 3.1.2. Impact of EVOH and Ormocer^®^ Coating on Tensile Properties

**Figure 4:**
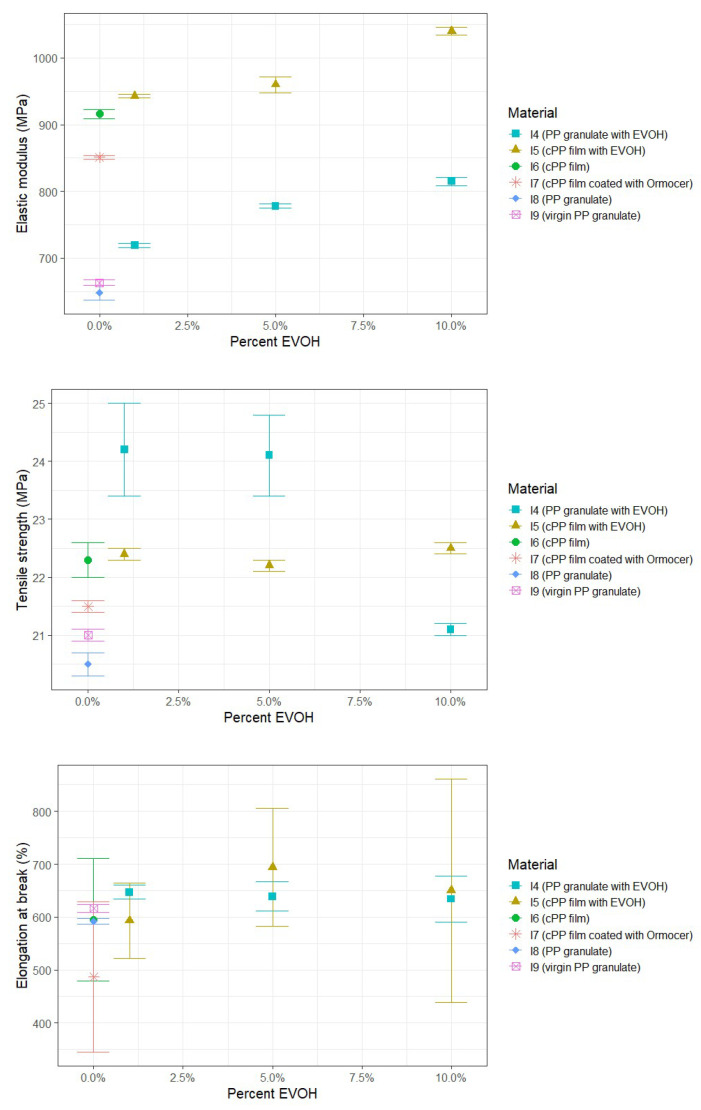
Results of tensile tests of PP containing varying amounts of EVOH as well as Ormocer^®^ coating after extrusion and injection molding.

The results of the tensile test for samples with EVOH and Ormocer^®^ can be seen in Figure 4. For comparison with I4 (PP granulate with EVOH), I8 (PP granulate) is a more relevant reference than I9 (virgin PP granulate) since it shares the same processing history. I6 (cPP film) serves as a reference for I5 (cPP film with EVOH) and I7 (cPP film coated with Ormocer^®^).

The results indicate that the elastic modulus increases with increasing EVOH content in both the cPP film and PP granulate. Additionally, I4 (PP granulate with EVOH) shows a slight increase in tensile strength, averaging 12%, at EVOH contents of 1% and 5%, along with slight increases in elongation at break when EVOH is added. However, the tensile strength of I5 (cPP film with EVOH) was in the same range as the reference I6 (cPP film). In contrast, the cPP film coated with Ormocer^®^ exhibits a slight decrease in elastic modulus, averaging around 7%, a decrease in tensile strength of around 4%, and a reduction in elongation at break of approximately 18% compared to I6 (cPP film). It is also noted that the standard deviations in elongation at break for the cPP film samples are generally very high compared to the granulated materials.

### 3.2. Determination of Impact Properties (Charpy)

#### 3.2.1. Impact of Labels on Charpy Impact Properties

**Figure 5:**
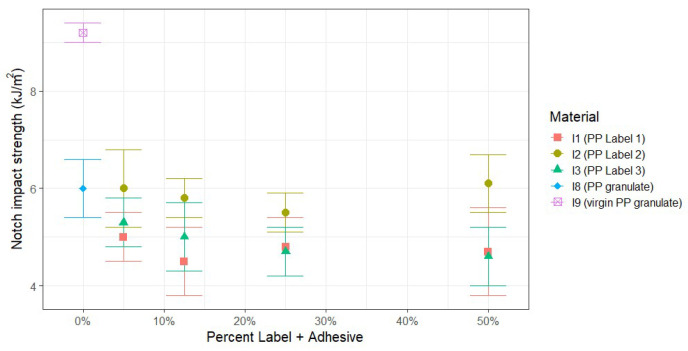
Results of the notched impact strength of PP containing varying amounts of labels after extrusion and injection molding.

The results in Figure 5 indicate that I8 (PP granulate) exhibits an average of 35% lower notched impact strength compared to I9 (virgin PP granulate). This implies that a single extrusion process of the virgin material already affects this parameter. As mentioned in Section 3.1.1, I8 (PP granulate) is a more relevant reference for comparison with I1-I3 because it shares the same processing history of extrusion and injection molding. The analysis shows that the notched impact strength of PP labels with adhesive type 2 (rubber-based) and the printing ink system used does not substantially deviate from the reference I8 (PP granulate), regardless of the content of the PP labels. The other two adhesive types, 1 (acrylate-based) and 3 (UV acrylate-based), are on average comparable to each other in terms of notched impact strength, and only slightly below the value of PP without any labels, i.e., I8 (PP granulate).

#### 3.2.2. Impact of EVOH and Ormocer^®^ Coating on Charpy Impact Properties

**Figure 6:**
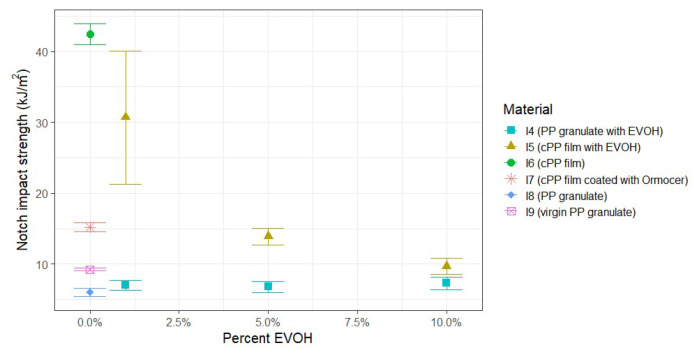
Results of notched impact strength of PP containing varying amounts of EVOH as well as Ormocer^®^ coating after extrusion and injection molding.

Analysis of the PP granulate materials shown in Figure 6, containing 1%, 5% and 10% EVOH, i.e., I4 (PP granulate with EVOH), reveals that the notched impact strength is similar compared to I8 (PP granulate) after single-cycle recycling, largely independent of the EVOH content. This indicates that the proportion of the EVOH type used, ranging from 1% to 10%, has minimal influence on the notched impact strength when combined with the PP granulate. In contrast, the cPP film used in the tests exhibits a marked decline in notched impact strength with the addition of EVOH, with reductions ranging from an average of 28% at 1% EVOH to 77% at 10% EVOH. The cPP film coated with Ormocer^®^ also showed a reduction in notched impact strength compared to the uncoated cPP film I6, which is approximately equivalent to the value of the cPP film with 5% EVOH.

### 3.3. Determination of MFR

The MFR measurements for PP, including those with added labels, EVOH, and Ormocer^®^ coating, are summarized in Table 3. The MFR of the recyclate R8 (PP granulate) and the sample containing PP label with adhesive type 3 do not considerably differ on average, from that of the virgin PP granulate. The recyclates containing PP labels with adhesive types 1 and 2 exhibit a slightly higher MFR on average, with the PP label compound containing adhesive type 2 showing a high standard deviation. Recyclate R4, consisting of PP granulate with 10% EVOH content, shows only a slight increase in MFR compared to R8 (PP granulate). The cPP film with Ormocer^®^ coating exhibits a minimal increase in MFR compared to the uncoated cPP film R6. An EVOH content of 10% results in an increase in the MFR of the cPP film from 4.4 to 5.7 g/10 min.

### 3.4. Determination of OIT

The investigation of the OIT for the recyclate R8 (PP granulate), as well as the compounds containing labels R1–R3, revealed only minor variations compared to the virgin PP granulate (Table 4). Additionally, the application of Ormocer^®^ coating on cPP films exhibited a negligible impact on the OIT values compared to R6 (cPP film). Notably, R5 (cPP film with EVOH) demonstrated an onset temperature approximately 10 °C higher than the cPP film without EVOH. Similarly, R4 (PP granulate with EVOH) showed an onset temperature around 20 °C higher than the PP granulate without EVOH. These results suggest an enhanced oxidation stability of PP in the presence of EVOH after extrusion, compared to PP compounds without EVOH.

### 3.5. Determination of Differences in Odor

The simulated recycling of PP did not affect the perceived odor, as both R8 (PP granulate) and virgin PP received the same grade. The grading system (0–4), detailed in Table 2, ranges from 0, indicating no perceptible odor difference, to 4, indicating a strong odor difference. The results presented in Table 5 show that all three label types and the Ormocer^®^ coating differed notably in terms of odor from both R8 (PP granulate) and the virgin PP granulate. Although the compounds containing EVOH also exhibited odor deviations, these differences were less pronounced compared to those associated with the labels and the Ormocer^®^ coating.

The differences in odor of the tension rods after further injection molding of the recyclate are listed in Table 6. All three PP labels and the dilutions, as well as the cPP film coated with Ormocer^®^, showed strong odor deviations, as already noticed for the recyclate. In addition, there was also a strong odor deviation in the PP granulate with 10% EVOH admixture. Weak odor deviations were observed in the cPP films with 1, 5 and 10% EVOH as well as the PP granulate with 1 and 5% EVOH. The additional injection-molding processing step caused increased differences in odor of I6 (cPP film), I8 (PP granulate) and I9 (virgin PP granulate) compared to the corresponding recyclates R6, R8 or virgin PP granulate.

## 4. Discussion

### 4.1. Challenges in Meeting EU Packaging Waste Regulations

The new EU’s PPWR envisages 10% recycled content for contact-sensitive packaging made of plastic materials other than PET from 2030, and 25% from 2040, with the exception of single-use beverage bottles [8]. In this study, however, the recycled content was set at 100%, exceeding the levels specified in the regulation as well as current industrial practices. This approach was adopted to simulate a worst-case scenario. Key properties such as mechanical performance, OIT, MFR and deviations of odor were tested to assess the feasibility and implications of using high levels of recycled material.

This approach provides a foundation for understanding the potential challenges that may arise from increased recycled content. Mechanical properties are crucial for determining the structural integrity and durability of packaging [37]. MFR, as a processing characteristic, could impact the ease and efficiency of manufacturing [37]. OIT plays a role in assessing the material’s stability during processing, with lower OIT values potentially indicating faster degradation during recycling [38]. Meanwhile, odor, an important factor for consumer acceptance, could be influenced by the presence of contaminants or degradation in recycled materials [39]. Behavior of PP alone during recycling has been studied with a few recent publications summarized in Table 7. The studies found that molecular weight, molecular weight distribution, and crystallinity influence various parameters, including those studied in this work. Not within the scope of this study was the safety aspect of PP recycling, which has been addressed in previous studies [40,41].

### 4.2. Mechanical Properties of Recycled PP

Tensile properties of PP before and after single-cycle recycling remained comparable, as previously reported [46,47]. Recycling reduces the impact strength of PP drastically as shown in earlier reports [47]. However, it should be noted that the cPP film tested exhibited exceptionally high notched impact strength in its initial state due to its application in stand-up pouches designed for high puncture resistance.

The tensile tests showed that the elastic modulus, tensile strength, and elongation at break of recyclates with varying label content were affected by both the label/adhesive system and the label concentration. No adverse effects were observed in samples with 5% and 12.5% PP label content. In contrast, a remarkable reduction in mechanical properties was noted at 50% label content, with the most substantial decline in elongation at break occurring in samples with PP labels using adhesive type 3 (UV acrylate-based). The addition of labels led to only a slight decrease in notched impact strength. The influence of labels on plastic recycling has received limited attention in the literature, with only a few recent studies addressing this issue. Schlossnikl et al. studied the mechanical properties of PP labels blended with virgin PP in mechanical recycling differentiating between pre-consumer and post-consumer labels [48]. The concentration of the labels varied between 10 and 50 wt.% with increments of 10%. Elastic modulus and elongation at break both declined upon addition of pre-consumer or post-consumer labels. In addition, yield stress was reported, which also declined with increasing label concentration. Tensile impact strength was not affected by the addition of labels in their study. The difference to our study could be explained by the fact that Schlossnikl et al. blended the labels with virgin PP whereas in our study labels were combined with recycled polymers. Traxler et al. studied the effects of various contaminants on the mechanical properties of PP recyclates, where one of them was in-mold labels with a content of 1% in a PP matrix [49]. It was found that in-mold labels were well dispersed in the matrix and even increased intermediate-rate tensile strength and tensile impact strength. This observation was attributed to the higher ductility of the in-mold labels compared to the PP matrix. In addition, strain at break increased from 6.4% ± 0.9 to 8.8% ± 2.4 upon addition of labels. The results of this study, along with the existing literature, implies that labels can negatively impact mechanical properties depending on their concentration [42]. Low concentrations, however, do not appear to cause any adverse effects [42].

The addition of EVOH either did not affect, or enhanced the tensile properties of the PP recyclates, particularly increasing the elastic modulus. The combination of PP and EVOH has been previously studied [50,51,52,53]. Faisant et al. prepared blends of PP and EVOH using an internal batch mixer as well as extrusion with subsequent drawing [52]. Elastic modulus of the blend prepared by batch mixing was not influenced by the addition of EVOH up to 25 vol.%. However, yield stress and elongation at break both decreased when concentration of EVOH was increased. It was suggested that decrease in elongation at break was due to the presence of large EVOH particles in the PP matrix, which may interrupt the PP polymer network and cause breakage at small strain. However, the blends containing 10 vol.% EVOH prepared by drawing between rolls after extrusion showed very similar mechanical properties compared to the PP with the same processing history not containing EVOH. This along with more studies implies that the processing technique or processing parameters greatly effect the distribution of EVOH in the PP matrix [51]. This, for examplecould be further studied using morphological characterization techniques such as scanning electron microscopy [54]. It should be noted that although PP and EVOH are compatible, they are not miscible [55]. Hence properties of PP/EVOH blends may be further enhanced through the use of compatibilizers [50,51]. In the case of recycled cPP films, a substantial drop in notched impact strength was observed in the presence of EVOH. This is a special situation because the cPP films were designed to have very high puncture resistance. This gives the film a high notched impact strength, which appears to be reduced by EVOH. Similarly, Wieczorek et al.’s work on the mechanical recycling of PP/EVOH/PP packaging containers revealed that, with the exception of impact strength, which was lower for the sample containing EVOH, mechanical properties were unaffected by recycling [53].

Ormocer^®^ has been reported to provide good abrasion resistance when applied to various polymer substrates [17]. Its impact on the mechanical properties in terms of tensile properties or impact strength to the best of our knowledge remains unexamined in scientific research. In our study we found that the tensile properties as well as the impact strength of the recyclate declined when cPP film was coated with Ormocer^®^ compared to the uncoated cPP film. The most likely explanation is that the Ormocer^®^ formed particles during extrusion and injection molding, which acted as defects during the tests as evidenced by black spots observed in the tension rods of sample number 7 (Figure 2c).

### 4.3. MFR of Recycled PP

Single-cycle recycling, as conducted in this study, did not affect the MFR of PP, which is consistent with previous results [56]. Nevertheless it has been reported that further recycling steps increased the MFR of PP considerably due to degradation by a chain scission mechanism [46,56,57,58]. Recyclates R1 and R2, containing 50% of two distinct labels and 50% PP, exhibited a higher MFR compared to the PP recyclate. In contrast, R3, which included a different type of label, showed no impact on the MFR. EVOH increased the MFR in both PP granulate and cPP film. *Touil* et al. reported that adding EVOH to a PE recyclate increased the MFR, which was anticipated since the EVOH used had a higher MFR than the PE [59].

Formulas like Arrhenius or Cragoe are used to estimate the final blend’s MFR based on the individual components’ MFRs and their proportions [60]. However, in the present study the exact MFR values of the cPP film and the EVOH granulate were not available. This information would have been necessary for a prediction, as both materials may exhibit broad viscosity ranges. For example, typical EVOH grades span from approximately 1.6 g/10 min to 12 g/10 min [61,62], depending on ethylene content and molecular weight. Likewise, virgin PP granulates used in injection-molding applications also cover a wide spectrum: Homopolymers typically range from 12 to 50 g/10 min, block copolymers from 3.5 to 100 g/10 min, and random copolymers from 13 to 80 g/10 min [63]. Ormocer^®^ has a minimal effect on the MFR of the PP recyclate. The influence of this factor has not yet been investigated in existing studies. In general, the MFR is highly dependent on the respective application, for instance PP grades with a low MFR are typically used for blow molding and extrusion, while high MFR grades are used for injection molding [64]. Consequently, the MFRs measured in this study are on the lower end for injection-molding applications. It is important to consider that various factors can influence the MFR of post-consumer recyclates. For instance, the presence of different PP and PE types in flexible packaging or variations in material mix (PE and PP content) after sorting can impact the results [65].

### 4.4. Thermo-Oxidative Stability of Recycled PP

The OIT measurements revealed that the extrapolated onset temperature of the virgin PP was similar to the recycled material R8 meaning that they have a similar thermos-oxidative stability. This observation is contradictory to the findings of Camacho and Karlsson which measured a sharp decrease in OIT after the initial two extrusion steps [66]. However, it is important to note that thermo-oxidative stability is heavily influenced by factors such as the presence of (inherent) stabilizers such as antioxidants or the filler content in the PP used [38,67,68]. Additionally, Camacho and Karlsson carried out their experiments with recycled PP, which may explain the observed variations in OIT values [38,66]. A slightly higher oxidation stability of PP in the presence of EVOH after processing was observed. Predicting OIT values of blends using the OIT values of the individual components can be performed using Non-Linear Regression approaches or Partial Least Squares regression [69]. The labels consisting of PP, color pigments and adhesives as well as the Ormocer^®^ coating did not influence the thermo-oxidative stability of the PP recyclates. The thermal stability of various pigments as measured by thermogravimetric analysis under an inert atmosphere was similar or higher compared to PP in previous work [70,71]. For the adhesives, thermal stability was found to be only slightly lower or similar to the PP [72,73,74]. According to the literature, none of the label components exhibit substantial lower thermal stability than PP, which may explain why no adverse effect of the labels on the OIT was observed. It has been mentioned before that the Ormocer^®^ formed particles during extrusion and injection molding, suggesting a lower thermal stability of Ormocer^®^. Yet this was not reflected in the results of the OIT measurements.

### 4.5. Odor Characteristics of Recycled PP

The extrusion of the PP granulates did not cause a detectable difference in odor, i.e., there was no difference in odor between R8 (PP granulate) and virgin PP granulate. However, after the injection-molding odor deviations of both I8 (PP granulates) and I9 (virgin PP granulate) could be detected. Thermal, thermo-mechanical, and thermo-oxidative degradation of both the polymer and its additives can produce lower molecular weight and lower boiling point compounds that volatilize, which contribute to the odor in recycled PP [75]. The differences in odor among recyclates with labels are likely due to the adhesives [76]. The odor deviations in the recyclate containing Ormocer^®^ are potentially result from its low thermal stability, as indicated by the presence of localized black impurities in the tension rods of sample number 7 (Figure 2c). A reduction in odor impairment after a recycling process could be possible using an extruder with integrated vacuum degassing and/or special deodorization processes such as steam stripping and PEG extraction [77] for colored non-food packaging and technical, high-quality components. For sensitive applications, however, such as contact-sensitive components, it is essential to identify these volatile substances, evaluate their impact on organoleptic properties, and assess their potential health effects [78].

## 5. Conclusions

This study investigates the impact of EVOH, Ormocer^®^ coating, and three printed labels with varying adhesive systems on the mechanical properties of recyclates, testing for injection-molding applications. A 100% recycled content was used, surpassing the targets set by the EU Packaging and Packaging Waste Regulation (PPWR), to evaluate the effects of high recycled material incorporation. Additionally, the study examines key parameters such as MFR and OIT, alongside an analysis of odor variations. The main findings are summarized as follows:

- Single-cycle recycling of virgin PP without colors, adhesives and EVOH had little impact on the tensile properties, but the impact strength decreased notably.
- Low concentrations of PP labels (5–12.5%) had a minimal effect on the mechanical properties of the recyclates, whereas at 50% label content, a marked reduction in mechanical performance was observed, depending on the adhesive used.
- EVOH increased the elastic modulus of the recyclates; however, a considerable reduction in impact strength was observed in recycled cPP films containing EVOH.
- Ormocer^®^ coating led to a decrease in both tensile and impact strength, likely due to particle formation during processing which acted as defects.
- Single-cycle recycling of virgin PP without colors, adhesives and EVOH did not affect the MFR of PP.
- Labels had varying effects on MFR depending on adhesive type, while the addition of EVOH increased MFR. Ormocer^®^ had minimal impact on MFR, though its influence remains underexplored.
- Single-cycle recycling of virgin PP without colors, adhesives and EVOH did not affect the OIT.
- Slightly higher oxidation stability was observed in PP recyclates containing EVOH.
- PP recyclates containing labels as well as the recycled Ormocer^®^-coated cPP film showed pronounced differences in odor compared to both the PP recyclate and the virgin PP granulate.

While some changes in properties were observed, the overall results are promising for advancing recycling practices and the successful integration of recycled materials.

## Figures and Tables

**Table 1:** Composition of test specimen for tensile test and Charpy Impact test. For production of the test specimen I1–I9 via injection-molding recyclates R1–R8 as well as virgin PP granulate were used. * Dilution with R8 (PP granulate). ** Dilution with R6 (cPP film).

Test Specimen	Name	Composition		Derived From
		PP	Label + Adhesive	
1	I1 (PP label 1)	50%	50%	R1 diluted with R8
1a *		75%	25%	
1b *		87.5%	12.5%	
1c *		95%	5%	
2	I2 (PP label 2)	50%	50%	R2 diluted with R8
2a *		75%	25%	
2b *		87.5%	12.5%	
2c *		95%	5%	
3	I3 (PP label 3)	50%	50%	R3 diluted with R8
3a *		75%	25%	
3b *		87.5%	12.5%	
3c *		95%	5%	
		PP	EVOH	
4	I4 (PP granulate with EVOH)	90%	10%	R4 diluted with R8
4a *		95%	5%	
4b *		99%	1%	
5	I5 (cPP film with EVOH)	90%	10%	R5 diluted with R6
5a **		95%	5%	
5b **		99%	1%	
6	I6 (cPP film)	100% PP		R6
7	I7 (cPP film coated with Ormocer^®^)	100% PP with Ormocer		R7
8	I8 (PP granulate)	100% PP		R8
9	I9 (virgin PP granulate)	100% PP		R9 = virgin PP granulate

**Table 2:** Grading System for Odor Intensity Differences according to DIN EN ISO 5495.

Grade of Difference in Odor Intensity	Description
0	No perceptible difference in odor
1	Barely perceptible difference in odor
2	Mild difference in odor
3	Noticeable difference in odor
4	Strong difference in odor

**Table 3:** Results of the MFR measurements on the recyclates as well as the virgin PP granulate.

Material (Granulate)	MFR (g/10 min)
R1 (PP label 1)	7.1 ± 0.1
R2 (PP label 2)	6.9 ± 1.4
R3 (PP label 3)	6.1 ± 0.6
R4 (PP granulate with EVOH)	6.4 ± 0.1
R5 (cPP film with EVOH)	5.7 ± 0.0
R6 (cPP film)	4.4 ± 0.1
R7 (cPP film coated with Ormocer^®^)	4.6 ± 0.0
R8 (PP granulate)	5.9 ± 0.1
virgin PP granulate	6.2 ± 0.2

**Table 4:** Extrapolated onset temperatures of the OIT (dynamic) using DSC on the recyclates as well as the virgin PP granulate.

Material (Granulate)	Extrapolated Onset Temperature (°C)
R1 (PP label 1)	221
R2 (PP label 2)	217
R3 (PP label 3)	221
R4 (PP granulate with EVOH)	237
R5 (cPP film with EVOH)	236
R6 (cPP film)	226
R7 (cPP film coated with Ormocer^®^)	224
R8 (PP granulate)	217
virgin PP granulate	218

**Table 5:** Differences in odor intensity of the recyclates as well as the virgin PP granulate. The grading system (0–4) reflects the perceived differences in odor intensity: 0 indicates no perceptible difference, while 4 indicates a strong difference in odor (for details see Table 2).

Material (Granulate)	Median of Difference in Odor Intensity
R1 (PP label 1)	3.0
R2 (PP label 2)	4.0
R3 (PP label 3)	3.75
R4 (PP granulate with EVOH)	2.5
R5 (cPP film with EVOH)	2.25
R6 (cPP film)	2.0
R7 (cPP film coated with Ormocer^®^)	3.5
R8 (PP granulate)	1.0
virgin PP granulate	1.0

**Table 6:** Differences in odor of injection-molded tension rods. The grading system (0–4) reflects the perceived differences in odor intensity: 0 indicates no perceptible difference, while 4 indicates a strong difference in odor (for details see Table 2). * Dilution with R8 (PP granulate). ** Dilution with R6 (cPP film).

Test Specimen	Name	Composition		Median of Difference in Odor Intensity
		PP	Label + Adhesive	
1	I1 (PP label 1)	50%	50%	3.5
1a *		75%	25%	4.0
1b *		87.5%	12.5%	3.5
1c *		95%	5%	3.0
2	I2 (PP label 2)	50%	50%	4.0
2a *		75%	25%	4.0
2b *		87.5%	12.5%	3.25
2c *		95%	5%	3.5
3	I3 (PP label 3)	50%	50%	3.0
3a *		75%	25%	4.0
3b *		87.5%	12.5%	3.5
3c *		95%	5%	2.5
		**PP**	**EVOH**	
4	I4 (PP granulate with EVOH)	90%	10%	4.0
4a *		95%	5%	2.25
4b *		99%	1%	2.25
5	I5 (cPP film with EVOH)	90%	10%	2.5
5a **		95%	5%	2.5
5b **		99%	1%	2.75
6	I6 (cPP film)	100% PP		2.25
7	I7 (cPP film coated with Ormocer^®^)	100% PP with Ormocer^®^		3.75
8	I8 (PP granulate)	100% PP		2.0
9	I9 (virgin PP granulate)	100% PP		1.75

**Table 7:** Summary of some studies on the properties of recycled PP versus virgin PP (Mw: molecular weight; TGA: thermogravimetric analysis; MFI/MFR: melt flow index/melt flow rate; DSC: differential scanning calorimetry; GPC: gel permeation chromatography; SEM: scanning electron microscopy; rPP: recycled polypropylene; TR: number of times the material could be recycled); ↑ increase; ↓ decrease; → results in.

References	Processing (How Recycling was Simulated)	Properties Studied	Main Findings
[42]	Twin-screw extruder–counter-rotating	Tensile, TGA, MFI, Impact strength	rPP mechanical properties ↓ vs. virgin PP Mixed PP waste is suitable only for thick-walled injection molding Impact strength highly variable → poor impact performance Heterogeneity of PP waste is important PP degrades quickly → limited recycle cycles
[43]	Co-rotating twin-screw extruder	MFR, DSC, Polarized Optical Microscopy, Tensile, GPC, SEM	Mw↓ and narrower distribution → chain scission (mainly long chains) MFR ↑ → lower melt viscosity Slower crystallization rate in rPP Tensile strength unchanged; slight decrease in Young’s modulus, increase in elongation at break → good recycling potential
[44]	Injection-molding machine; five different compositions of PP/rPP	Tensile, Flexural strength, Bending modulus, SEM	Yield strength and tensile strength constant Elongation at break ↑ Young’s modulus ↓ slightly Thermal degradation during reprocessing as well as chain scission Molecular weight ↓ Flexural strength and bending modulus ↓ rPP showed minimal mechanical deterioration overall
[45]	Injection-molding machine; multiple recycling cycles	MFI, Thermal behavior, Tensile	Tensile strength ↑ (best at TR3; decreased after TR4) Crystallinity and crystallization rate ↑ MFI ↑ with increasing recycling cycles

## Data Availability

The original contributions presented in this study are included in the article. Further inquiries can be directed to the corresponding authors.

## Supplementary Materials

The following supporting information can be downloaded at: https://www.mdpi.com/article/10.3390/polym17243332/s1, Table S1: Extrusion parameters for compounding and recyclate production. Speed 400 rpm, scroll: standard, outgassing: P4 open, torque: 55 Nm, pressure: 18 bar; Table S2: Injection molding parameter, injection pressure: 400 bar, back pressure: 200/250 bar, back pressure: 50 bar
