# Supplementary material for: Impact of EVOH, Ormocer® Coating, and Printed Labels on the Recyclability of Polypropylene for Packaging Applications
Source: Polymers (Basel). 2025 Dec 17;17(24):3332. doi: 10.3390/polym17243332 (PMC12737107; doi:10.3390/polym17243332)
Supplement: Supplementary file 1 [file polymers-17-03332-s001.zip › polymers-3973750-supplementary.pdf]

Supplementary Materials

Impact of EVOH, Ormocer® Coating, and Printed Labels on the Recyclability of Polypropylene for Packaging Applications

Romana Schmiedt [1], Michael Krainz [2], Katharina Tosic [1], Farshad Sharbafian [1], Simon Krauter [1,], Victoria Krauter [1], Martin Novak [1], Bernhard Rainer [*1], Michael Washüttl [2] and Silvia Apprich [1]

Institute of Packaging and Resource Management, Department Applied Life Sciences, University of Applied Sciences, 1100 Vienna, Austria; romana.schmiedt@hcw.ac.at (R.S), katharina.tosic@hcw.ac.at (K.T), farshad.sharbafian@hcw.ac.at (F.S), vt@hcw.ac.at (S.K.), martin.novak@hcw.ac.at (M.N), bernhard.rainer@hcw.ac.at (B.R.), silvia.apprich@hcw.ac.at (S.A)

[2] OFI - Österreichisches Forschungsinstitut für Chemie und Technik, Franz-Grill-Straße 5, Obj. 213, 1030 Vienna; michael.krainz@ofi.at (M.K), michael.washuettl@ofi.at (M.W)

* Correspondence: bernhard.rainer@hcw.ac.at; Tel.: +43 1 6066877 3583

Table S1. Extrusion parameters for compounding and recyclate production. Speed 400 rpm, scroll: standard, outgassing: P4 open, torque: 55 Nm, pressure: 18 bar.

Temperature zone	Temperature (°C)
Mass	186
Nozzle	185
Zone 10	190
Zone 9	200
Zone 8	205
Zone 7	210
Zone 6	220
Zone 5	230
Zone 4	230
Zone 3	230
Zone 2	190

Table S2 : Injection molding parameter, injection pressure: 400 bar, back pressure: 200/250 bar, back pressure: 50 bar

Temperature zone	Temperature (°C)
Tool	40
Nozzle	210
Zone 3	220
Zone 2	225
Zone 1	230
